# Evaluation of beta-propiolactone and AVL-ethanol inactivation protocols for highly pathogenic avian influenza virus in allantoic fluid and cell culture

**DOI:** 10.1128/aem.02144-25

**Published:** 2026-02-24

**Authors:** Natalia Pessoa, Nathalie Berube, Lauren Aubrey, Jill Van Kessel, M. Afzal Javed, Yan Zhou

**Affiliations:** 1 Vaccine and Infectious Disease Organization (VIDO), University of Saskatchewan 7235, Saskatoon, Saskatchewan, Canada; 2 Vaccinology and Immunotherapeutics Program, School of Public Health, University of Saskatchewan 248223, Saskatoon, Saskatchewan, Canada; 3 Department of Veterinary Microbiology, Western College of Veterinary Medicine, University of Saskatchewan 70399, Saskatoon, Saskatchewan, Canada; Centers for Disease Control and Prevention, Atlanta, Georgia, USA

**Keywords:** containment level 3 (CL3) laboratories, beta-propiolactone, effective inactivation of virus, highly pathogenic avian influenza H5N1

## Abstract

Highly pathogenic avian influenza (HPAI) H5N1 recently expanded its host range to include dairy cattle, and since March 2024, there have been over 1,000 cases reported in the United States. This circulation in dairy cows and resulting spillover to other mammals, including 41 documented human cases, raises the risk of reassortment and development of mutations that increase virus fitness in mammalian hosts. Research on HPAI is crucial, but due to its high pathogenicity, work with live virus must be conducted in containment level 3 laboratories. Strict biosafety procedures are essential when handling virus-containing materials to ensure safety. In this report, we explore options for inactivating the virus to enable safe work in containment level 2 (CL2) laboratories, which allow for more efficient work. We tested beta-propiolactone as an inactivation agent for HPAI in cell culture media and allantoic fluid from eggs. We also tested whether Qiagen Buffer AVL with 95% ethanol could inactivate the virus in allantoic fluid. We report that 0.1% and 0.2% beta-propiolactone and Buffer AVL with ethanol were successful in completely inactivating the HPAI in cell culture media and allantoic fluid. These inactivation methods allow safe transfer of samples to CL2 laboratories and facilitate downstream application such as vaccine testing, ELISA, qPCR, and RNA sequencing.

**IMPORTANCE:**

Due to the zoonotic potential of highly pathogenic avian influenza (HPAI) H5N1 virus and its highly pathogenic features, stringent biosafety measures are essential when handling virus-containing material, and work is required to be performed in containment level 3 laboratories. If any downstream work pertaining to the inactivated virus will be performed in containment level 2 laboratories, validation of complete inactivation of the virus is required, as incomplete inactivation poses a serious risk of laboratory-acquired infections and environmental release. Our study established and validated protocols of complete inactivation of HPAI H5N1 virus in allantoic fluid as well as in tissue culture medium by beta-propiolactone and AVL plus 95% ethanol.

## INTRODUCTION

Wild aquatic birds are the natural reservoir for influenza A virus, and they can be infected with H1–H16 influenza A subtypes, which typically cause mild symptoms in these wild waterfowl (1, 2). While most strains also cause mild disease in poultry, subtypes with H5 or H7 hemagglutinins (HA) have been shown to evolve when circulating in poultry, causing higher disease severity with mortality rates of up to 100%. These strains are classified as highly pathogenic avian influenza (HPAI) based on their pathogenicity in chickens (1).

In 1996, a new lineage of HPAI was detected in Guangdong Province, China, in domestic geese (Gs/Gd)(1). HPAI outbreaks have historically been limited to domestic poultry, but this Gs/Gd lineage spilled back over into wild migratory waterfowl and spread globally (3). This dissemination led to the diversification of the lineage through antigenic drift and shift. Subclade 2.3.4.4b emerged in 2020 and has gained global dominance, causing high morbidity and mortality in wild and domestic birds and spilling over into several mammalian species (3). Transmission of H5N1 to humans is typically through exposure to infected animals or contaminated environments, and sustained human to human transmission has not been observed (4). Since 2003, there have been 993 documented cases in humans, with a case fatality rate of 50%, though that may be an overestimate due to the fact that mild cases are less likely to be reported (4, 5).

In March of 2024, HPAI H5N1 virus was detected in dairy cattle in the United States and began to spread rapidly (2). To date, over 1,000 cases have been reported in 17 states (6). This circulation of HPAI in cows is unprecedented, as they are not typically productively infected with influenza. There have been 41 confirmed human cases linked to dairy cattle exposure, although this is likely underestimated (7). The virus has been transmitted to other mammalian species from cows, including raccoons and cats, as well as to domestic poultry and back into wild birds (8). Significant mortality was observed in these animals, including the cats that ingested raw milk. This circulation of HPAI in dairy cattle and resulting spillover into other mammalian species is of concern as it increases the risk of reassortment with seasonal influenza virus strains and the development of mammalian adaptations that could enable transmission between humans (9).

According to Canadian and U.S. regulation, naturally occurring H5N1 viruses are classified as risk group 3/biosafety level 3 (BSL-3) pathogens due to their high pathogenicity, and work with live virus must be undertaken in containment level 3 (CL3)/BSL-3 laboratories (10, 11). CL3 laboratory conditions are essential for ensuring safety when handling this high-risk pathogen; however, the decontamination protocols, physiological constraints, and the cumbersome PPE required for work in these facilities impose significant limitations on research. Additionally, CL3 laboratories are specialized and typically accessed by a restricted number of personnel. As a result, there is typically less equipment available to work with. Therefore, when work with live virus is not required, inactivation of certain sample types to allow safe work in containment level 2 (CL2) laboratories would allow for more efficiency.

Embryonated chicken eggs are an important tool in influenza research; they are susceptible to infection with avian influenza virus strains and can propagate them to high titers (12). Eggs are also the standard system for growing vaccine strains, including human seasonal influenza strains that are typically generated by combining the HA and neuraminidase (NA) genes from the strain of interest with the six internal genes from A/Puerto Rico/8/1934 (PR8) to facilitate viral replication in eggs (13, 14). Cell culture systems are also widely employed in laboratory research for virus propagation and virological assays and are used to grow some influenza viruses for vaccines, though to a lesser extent than eggs (15).

β-Propiolactone (BPL) is one of the most widely used chemicals for vaccine virus inactivation and has been commonly employed in the viral vaccine industry (16). HPAI virus inactivated with BPL could be used to test immunogenicity of vaccines in a CL2 environment, which would ease the burden on personnel handling the animals and processing samples and reduce material costs. BPL-inactivated virus could also serve as a capture antigen in an ELISA assay to detect vaccine-induced antibodies in samples collected prior to challenge, as well as in inactivated samples taken from animals challenged with HPAI. We previously reported various virus inactivation methods, including Buffer AVL with 95% ethanol inactivation of bovine H5N1 virus in supernatant from infected cells, milk, blood, and urine; Buffer RLT with 70% ethanol inactivation of bovine H5N1 virus in infected cell pellet, spiked milk, blood, urine, and tissue; and heat and Triton X-100 inactivation of serum and milk samples containing HPAI H5N1 (17). In this report, we investigate the effectiveness of BPL for inactivating allantoic fluid and cell culture media containing high titers of HPAI H5N1 virus. We also evaluated whether treatment with 95% ethanol and Buffer AVL from the Qiagen Viral RNA Extraction kit could effectively inactivate the virus in allantoic fluid, to enable sequencing or quantification of RNA from virus grown in eggs in CL2 conditions.

## MATERIALS AND METHODS

### Biosafety statement

Work with HPAI H5N1 was conducted in Vaccine and Infectious Disease Organization (VIDO) CL3, in compliance with the Canadian Biosafety Standard, 3rd ed., under the authority of the Public Health Agency of Canada and the Canadian Food Inspection Agency.

### Virus and cells

Madin-Darby canine kidney (MDCK; ATCC #CRL-2936) cells were maintained in minimal essential medium (MEM) (Sigma-Aldrich, M4655, St. Louis, MO, USA) containing 10% fetal bovine serum (Thermo Fisher Scientific, 16000-044, Ottawa, ON, Canada), and cells were kept in a humidified 5% CO_2_ incubator at 37°C. The HPAI H5N1 strain A/dairy cattle/Texas/24-008749-002/2024 was obtained from the United States Department of Agriculture and was propagated in MDCK cells in viral growth media (MEM containing 0.2% bovine serum albumin [Sigma-Aldrich, A7030, St. Louis, MO, USA]) with 1 μg/mL L-1-tosylamido-2-phenylethyl chloromethyl ketone-treated trypsin.

### Allantoic fluid harvest from embryonated eggs

Embryonated chicken eggs obtained from the University of Saskatchewan Poultry Teaching and Research Unit were incubated at 37°C for 11 days. Subsequently, 11-day-old embryonated eggs were inoculated with 10 TCID_50_ of HPAI H5N1 in the allantoic cavity. On day 3 post-infection, allantoic fluid was harvested, yielding a viral titer of 3.3 × 10⁸ TCID_50_/mL and a hemagglutination (HA) titer of 256.

### BPL treatment

To evaluate viral inactivation by BPL, 200 µL of either cell culture supernatant containing H5N1 (5.6 × 10⁷ TCID_50_/mL) or allantoic fluid containing H5N1 (3.3 × 10⁸ TCID_50_/mL; HA titer = 256) was treated with BPL to achieve a final concentration of 0.2% or 0.1%. If necessary, the pH was adjusted to neutral (pH 6–8) using 1 N NaOH. Samples were then incubated at 4°C for 16–18 h. To hydrolyze residual BPL, the samples were further incubated at 37°C for 2 h. Once more, pH was adjusted if needed, as previously described. Finally, the samples were centrifuged at maximum speed for 10 min, and the supernatant was collected for the assay.

### AVL-based inactivation of H5N1 in allantoic fluid

To evaluate viral inactivation using AVL Buffer from the QIAamp Viral RNA Mini Kit (Qiagen 52906, Toronto, ON, Canada), allantoic fluid harvested from embryonated chicken eggs infected with H5N1 virus was diluted 1:4. Then, 140 µL of the diluted sample was mixed with 560 µL of AVL Buffer and incubated at room temperature (RT) for 10 min. Subsequently, 560 µL of 95% ethanol was added and incubated at RT for an additional 10 min. Allantoic fluid harvested from PBS-inoculated embryonated chicken eggs (clean) was subjected to the same treatment as a control.

### Removal of cytotoxic buffers using centrifugal filters

The method is described in our previous publication (17). Briefly, samples in Buffer AVL/ethanol or treated with BPL were adjusted to a final volume of 4 mL with PBS and applied to the sample reservoir of an Amicon Ultra-4 Centrifugal Filter Unit (100 kDa, 4 mL) (Sigma-Aldrich, UFC810008, St. Louis, MO, USA). After centrifugation, the flow-through was discarded, and an additional 4 mL of PBS was added to the sample reservoir and centrifuged again. This process was repeated four times until 100–200 µL remained in the filter unit, and then this was used as an inoculum for testing residual infectivity of H5N1 particles.

### Testing for residual infectivity of H5N1 virus

MDCK cells were seeded in 12-well plates at 2 × 10⁵ cells per well and incubated overnight at 37°C with 5% CO_2_. Filtered inactivated samples were added onto the confluent MDCK monolayers and allowed to adsorb for 1 h at 37°C. After adsorption, the inoculum was removed and replaced with viral growth media. Cells were then incubated at 37°C with 5% CO_2_ and monitored daily for cytopathic effect (CPE) for 3 days. After the 3-day incubation, 250 µL of the supernatant was collected (round 1), centrifuged at 300 × *g* for 8 min to remove any cellular debris. While 100 µL was transferred to fresh MDCK cells for the next round of inoculation, 140 µL is used for RNA extraction and viral RNA determination. This process was repeated three additional times (rounds 2–4), for a total of four sequential passages. For positive control, cell culture-grown H5N1 virus and allantoic fluid harvested from HPAI-infected eggs were used to inoculate MDCK cells at an MOI of 0.001. Supernatants harvested on day 3 post-infection were diluted 1:1,000, and 100 µL was used to inoculate the cells in the next round. Mock-infected MDCK cells and MDCK cells inoculated with clean allantoic fluid were used as negative controls.

### Detection of viral RNA

RNA was extracted from the supernatant using the QIAamp Viral RNA Mini Kit (Qiagen, 52906, Toronto, ON, Canada), following the manufacturer’s protocol. Briefly, 140 μL of supernatant was mixed with 560 μL Buffer AVL and incubated at RT for 10 min, followed by the addition of 560 μL of 95% ethanol and another 10 min incubation. A portion (630 μL) of the lysate was loaded onto a QIAamp Mini spin and centrifuged; the column was then washed, and RNA was eluted in 50 μL elution buffer and stored at −80°C.

Quantitative RT-PCR was performed using 5 μL of RNA with the Luna Universal qPCR Kit (NEB, E3006L, Whitby, ON, Canada) on a StepOne Plus Real-Time PCR System (QuantStudio 3, Applied Biosystems) as described previously (17, 18). Primers and probe are targeting the influenza A virus matrix (M) gene: forward primer GGCCCCCTCAAAGCCGA, reverse primer CGTCTACGYTGCAGTCC, and probe 5′-(FAM)-TCACTGGGCACGGTGAGCGT-3′ (MGBNFQ) (IDT, Coralville, IA, USA). A standard curve was generated using RNA extracted from H5N1 virus with a defined titer and was used to interpolate TCID_50_ equivalents from Ct values.

### Determination of limit of detection

The limit of detection (LOD) experiment was conducted and determined according to published reports (19, 20). RNA was extracted from an H5N1 virus stock with a titer of 5.6 × 10⁷ TCID_50_/mL, and serially diluted to concentrations of 56, 28, and 5.6 TCID_50_ per reaction. Each dilution was tested in 20 technical replicates, and two independent experiments were performed on different days. The data presented here represent one of these experiments. Individual Ct values were recorded for each replicate, and the mean Ct value was calculated for each dilution. Ct values were recorded up to 40 cycles. Reactions with no detectable amplification were recorded as undetermined (Und).

## RESULTS

### Defining LOD and viral inactivation

**Fig 1:**
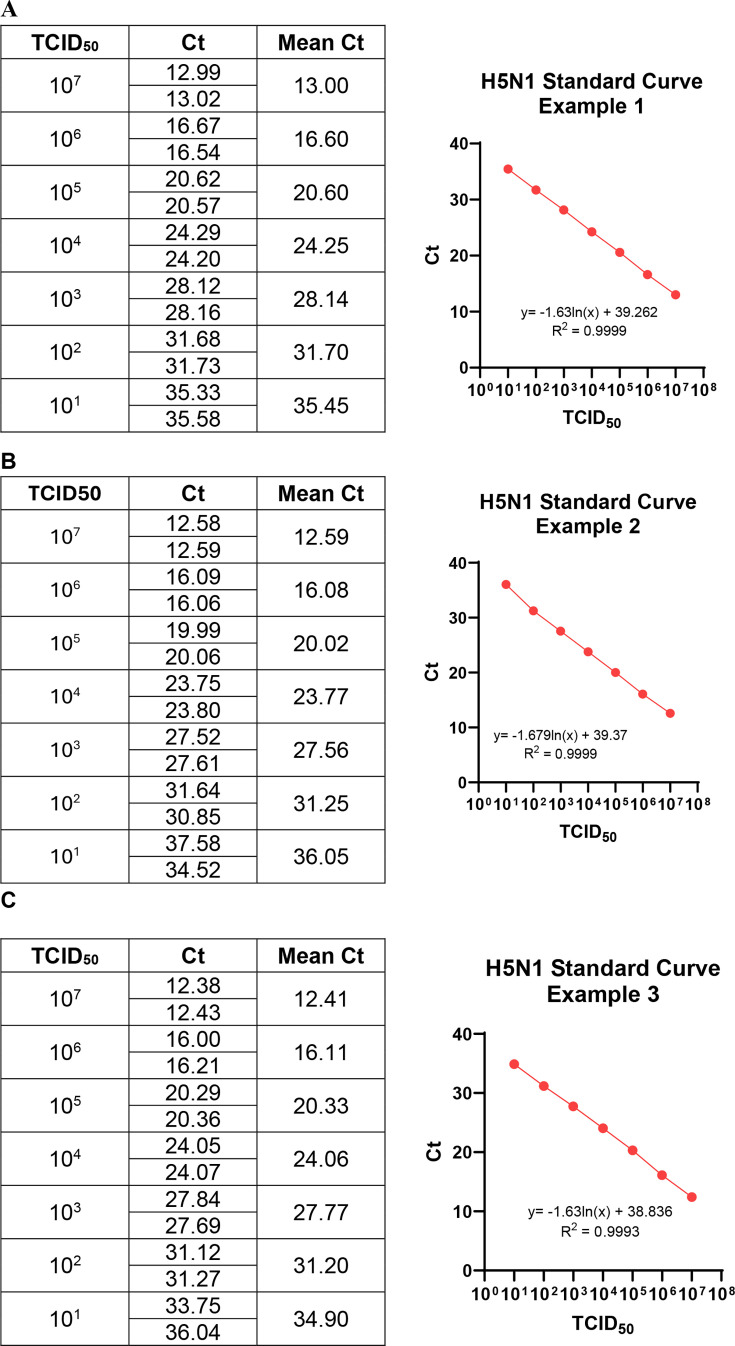
Standard curves for quantification of H5N1 viral RNA by RT-qPCR. RNA was extracted from minimal essential medium (MEM) spiked with 10^7^ TCID_50_ of HPAI H5N1 virus by Qiagen QIAamp Viral RNA Kit. RNA was diluted 10-fold with ddH_2_O. RT-qPCR was performed in duplicates. The mean value from duplicates was used to plot the standard curve. Three independent experiments are presented here (**A–C**).

We first established a standard curve to determine the correlation between infectious viral particle titer and Ct values obtained from RT-qPCR targeting the viral M gene. Although the standard curve on each plate varied slightly (Fig. 1), multiple rounds of experiments showed that RNA extracted from 10^7^ TCID_50_ of H5N1 yields a Ct value of 12.4–13.0, whereas 10 TCID_50_ of virus yields a Ct value of 34.9–36.1. We conducted further experiments to determine the LOD. RNA extracted from defined quantities of virus particles was subjected to RT-qPCR in 20 replicates. The LOD is defined as the lowest amount of analyte that yielded detectable amplification in at least 95% of replicates. As shown in Table 1, RNA extracted from 56 and 28 TCID_50_ was readily detected with 100% certainty, while RNA equivalent to 5.6 TCID_50_ was detected in 95% replicates. Therefore, we determined the LOD to be 5.6 TCID_50_, corresponding to a Ct value of 36. In our system, a Ct value >36.0 is considered negative for the presence of infectious virus. Effective viral inactivation was defined by the absence of CPE across four serial passages, accompanied by Ct values >36.0 and a progressive increase in Ct values, or consistent Ct values >36.0 over successive passages.

**TABLE 1:** Determination of the LOD^*a*^

Virus particle (TCID_50_)	Ct values of 20 replicates																				Ct mean	% positive
56	30.7	31.0	31.2	31.1	31.0	31.4	31.0	30.9	30.8	31.2	31.1	31.1	31.2	31.3	31.3	31.0	31.4	31.0	31.0	30.8	31.1	100
28	32.2	32.0	32.4	32.2	32.2	31.9	32.3	32.3	31.8	32.1	32.2	32.1	32.9	32.3	31.9	32.4	32.2	32.4	31.7	31.9	32.2	100
5.6	35.4	35.3	**Und**	36.4	35.7	36.6	36.1	35.7	35.5	35.8	35.1	35.3	36.1	37.3	35.5	36.3	36.6	38.0	35.7	35.3	36.0	95

^*a*^ The table shows the determination of the limit of detection (LOD) of the RT- qPCR assay using serial dilutions of RNA extracted from an H5N1 virus stock with a previously determined TCID_50_. Each dilution was tested in 20 technical replicates across two independent experiments. The table presents the results from one representative experiment. LOD was defined as the lowest viral quantity yielding ≥95% positive detection in RT-qPCR (Ct detects up to 40 cycles). Und, undetermined amplification.

### Viral inactivation by BPL

**Fig 2:**
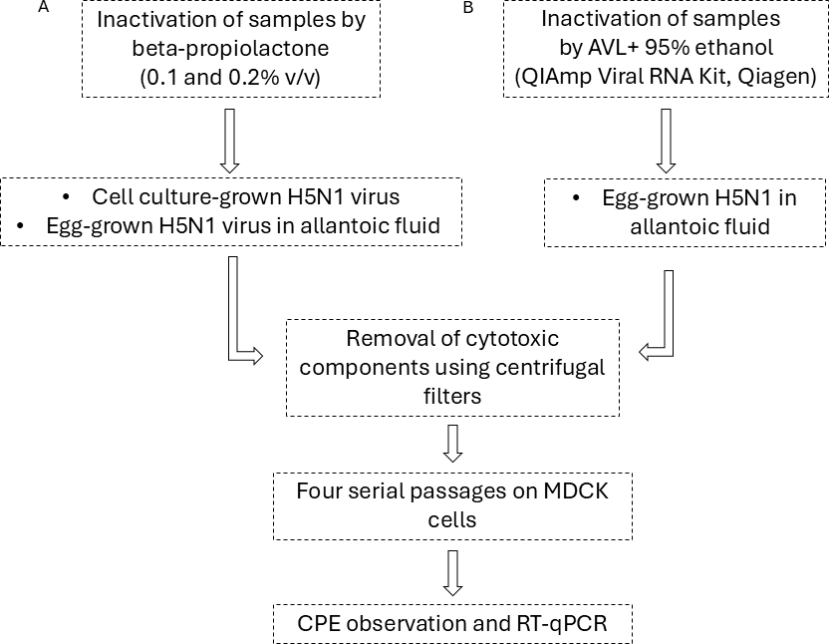
Schematic of the testing workflow. (**A**) Egg-grown H5N1 virus in allantoic fluid (3.3 × 10^8^ TCID_50_/mL) or tissue culture-grown H5N1 virus in minimal essential medium (MEM) (5.6 × 10^7^ TCID_50_/mL) were treated with beta-propiolactone (BPL) at 4°C for 16–18 h at final concentrations of 0.1% or 0.2% (vol/vol). (**B**) Egg-grown H5N1 virus in allantoic fluid was incubated with Buffer AVL for 10 min, followed by the addition of 95% ethanol and a second 10 min incubation. Samples treated with AVL + ethanol or BPL were then processed through a 100 kDa Amicon Ultra-4 centrifugal filter unit to remove cytotoxic components and subsequently passaged four times in MDCK cells to assess viral inactivation. On day 3 post-infection of each round, cytopathic effect (CPE) was evaluated, and supernatants were collected for viral RNA detection by RT-qPCR.

The workflow of this study is summarized in Fig. 2. To assess the ability of BPL to inactivate HPAI H5N1 virus grown in tissue culture and in embryonated chicken eggs, we have four test articles: Test Article #1 is the tissue culture-grown H5N1 treated with 0.1% BPL, Test Article #2 is the tissue culture-grown H5N1 treated with 0.2% BPL, Test Article #3 is the egg-grown H5N1 in allantoic fluid treated with 0.1% BPL, and Test Article #4 is the egg-grown H5N1 in allantoic fluid treated with 0.2% BPL. After filtration to remove the cytotoxic components, the samples have gone through four rounds of inoculation in MDCK cells. None of the BPL-treated samples induced CPE, independent of the concentration of BPL used or sample type (Table 2). Correspondingly, RT-qPCR analysis showed increasing Ct values or consistently high Ct values over passages, with no evidence of viral RNA amplification, indicating the absence of productive viral replication. For example, Test Article #1 showed an initial Ct of 24.9 on day 0 and a Ct of 32.9 on day 3 of the first passage, followed by Ct values ≥36.4 in subsequent rounds. Similar patterns were observed for Test Article #2. As for the egg-grown H5N1, allantoic fluid treated with 0.1% BPL (Test Article #3) showed Ct of 18.2 on day 0 and Ct of 28.0 on day 3 of round 1. The Ct value increased to 38.6 in round 2, 38.3 in round 3, and undetermined in round 4, indicating the effective inactivation of the virus. The same results were achieved for the allantoic fluid treated with 0.2% BPL (Test Article #4).

**TABLE 2:** Effectiveness of BPL treatment in inactivating tissue culture-grown H5N1 virus in MEM and egg-grown H5N1 virus in allantoic fluid^*a*^

Sample	Round 1					Round 2			Round 3			Round 4		
	Day 0		Day 3			Day 3			Day 3			Day 3		
	Ct	TCID_50_	Ct	TCID_50_	CPE	Ct	TCID_50_	CPE	Ct	TCID_50_	CPE	Ct	TCID_50_	CPE
Negative Control #1	37.4 ± 0.60	3.20E+00	Und		N	39.2 ± 1.10	6.05E−01	N	Und		N	Und		N
Negative Control #2	36.4 ± 0.10	5.95E+00	Und		N	Und		N	37.8 ± 3.09		N	Und		N
Test Article #1	24.9 ± 0.04	6.51E+03	32.9 ± 0.57	5.62E+00	N	39.4 ± 0.89	8.53E−02	N	36.4 + 5.14	2.23E+00	N	37.1 ± 1.64	1.65E+00	N
Test Article #2	25.8 ± 0.28	3.95E+03	35.4 ± 1.16	7.14E−01	N	Und		N	38.6 + 0.26	6.81E−01	N	37.8 ± 0.39	2.35E+00	N
Test Article #3	18.2 ± 0.00	4.11E+05	28.0 ± 0.06	6.36E+01	N	38.6 ± 0.23	5.72E−01	N	38.3 + 2.36	1.91E−01	N	Und		N
Test Article #4	22.7 ± 0.06	2.57E+04	32.7 ± 0.01	3.49E+00	N	38.3 ± 0.23	1.07E+00	N	Und		N	37.0 ± 1.33	1.61E+00	N
Positive Control #1	24.6 ± 0.05	8.19E+03	10.9 ± 0.06	3.54E+07	Y	11.0 ± 0.42	2.15E+07	Y	11.6 ± 1.02	1.75E+07	Y	10.6 ± 0.21	3.44E+07	Y
Positive Control #2	28.4 ± 0.02	7.90E+02	12.9 ± 0.65	1.08E+07	Y	10.1 ± 0.23	3.72E+07	Y	11.1 ± 0.52	2.21E+07	Y	10.9 ± 0.46	2.82E+07	Y

^*a*^ The table presents Ct values, converted virus quantity in TCID_50 _and cytopathic effect (CPE) observations recorded on indicative days for each sample. Negative Control #1, MDCK cells inoculated with MEM; Negative Control #2, MDCK cells inoculated with clean allantoic fluid; Test Article #1, tissue culture-grown H5N1 treated with 0.1% BPL; Test Article #2, tissue culture-grown H5N1 treated with 0.2% BPL; Test Article #3, egg-grown H5N1 in allantoic fluid treated with 0.1% BPL; Test Article #4, egg-grown H5N1 in allantoic fluid treated with 0.2% BPL; Positive Control #1, MDCK cells infected with cell-grown H5N1 virus; Positive Control #2, MDCK cells infected with egg-grown H5N1 virus. Each test article was inoculated into cell cultures in duplicate, and RT-qPCR was performed in technical duplicate. Ct values represent the mean of two technical replicates for each of two biological replicates. CPE is indicated as “Y” (yes) or “N” (no).

As controls, MDCK cells were infected with either cell-grown H5N1 virus (Positive Control #1) or egg-grown H5N1 virus (Positive Control #2), both at MOI = 0.001. Cells showed complete CPE on day 3 of each round of infection, and Ct values consistently decreased across subsequent rounds of infection, confirming robust viral replication. As negative controls, MDCK cells were infected with MEM (Negative Control #1) or allantoic fluid harvested from PBS-inoculated embryonic eggs (Negative Control #2) did not show any CPE and remained high Ct values (>36.4) throughout the four rounds of passages.

### Viral inactivation by Buffer AVL + 95% EtOH

To evaluate the inactivation capacity of the Qiagen QIAamp Viral RNA Kit during RNA extraction from egg-grown H5N1 in allantoic fluid, H5N1-containing allantoic fluid was treated with Buffer AVL and 95% ethanol (Test Article #5). During four serial passages in MDCK cells, no CPE was observed on day 3 post-inoculation in any of the four rounds (Table 3), indicating effective viral inactivation. RT-qPCR analysis of culture supernatants revealed initially detectable levels of viral RNA, with a Ct value of 30.6 on day 0 and increased to 35.3 on day 3 in round 1, indicating that the detected RNA represents residual input material rather than newly synthesized viral genomes. The Ct values steadily remained >37.2 across the subsequent rounds, indicating no evidence of viral replication.

**TABLE 3:** Effectiveness of AVL Buffer + 95% ethanol treatment for inactivation of H5N1 in allantoic fluid^*a*^

Sample	Round 1					Round 2			Round 3			Round 4		
	Day 0		Day 3			Day 3			Day 3			Day 3		
	Ct	TCID_50_	Ct	TCID_50_	CPE	Ct	TCID_50_	CPE	Ct	TCID_50_	CPE	Ct	TCID_50_	CPE
Test Article #5	30.6 ± 0.09	2.03E+02	35.3 ± 1.54	8.30E−01	N	Und		N	37.2 ± 3.91	7.50E−01	N	37.5 ± 0.41	5.41E−01	N
Negative Control #3	36.7 ± 0.35	4.91E+00	Und		N	Und		N	Und		N	Und		N
Negative Control #4	36.4 ± 0.10	5.95E+00	Und		N	Und		N	37.8 ± 3.09	1.79E+00	N	Und		N
Positive Control #2	28.4 ± 0.02	7.90E+02	12.9 ± 0.65	1.08E+07	Y	10.1 ± 0.23	3.72E+07	Y	11.1 ± 0.52	2.21E+07	Y	10.9 ± 0.46	2.82E+07	Y

^*a*^ The table presents Ct values, converted virus quantity in TCID _50_ and cytopathic effect (CPE) observations recorded on indicative days for each sample. Test Article #5, egg-grown H5N1 in allantoic fluid was treated with Buffer AVL and 95% ethanol; Negative Control #3, MDCK cells inoculated with AVL-treated clean allantoic fluid; Negative Control #4, MDCK cells inoculated with untreated clean allantoic fluid; Positive control #2M, MDCK cells inoculated with untreated egg-grown H5N1 in allantoic fluid. Each test article was inoculated into cell cultures in triplicate, and RT-qPCR was performed in duplicate. Ct values represent the mean of duplicate measurements of biological duplicates with standard deviation. CPE is indicated as “Y” (yes) or “N” (no).

As control, MDCK cells inoculated with AVL-treated clean allantoic fluid (Negative Control #3) and MDCK cells inoculated with untreated clean allantoic fluid (Negative Control #4) showed no CPE, and Ct values are >36.4 in all rounds. In contrast, MDCK cells inoculated with untreated H5N1 containing allantoic fluid (Positive Control #2) consistently exhibited strong CPE and high viral loads, as indicated by consistently low Ct values: 12.9 at round 1, day 3; 10.1 at round 2; 11.1 at round 3; and 10.9 at round 4 (Table 3), confirming active viral replication.

## DISCUSSION

In this report, we tested viral inactivation of HPAI H5N1 harvested from egg allantoic fluid and cell culture media using 0.1% and 0.2% BPL. BPL causes the alkylation of guanosine bases in viral RNA, interfering with viral transcription, as polymerases misread the modified guanosines as adenosine (21). BPL can also react with certain amino acids, which may also have an effect on infectivity (21, 22). After treatment with BPL, we found that neither sample type induced cytopathic effects in MDCK cells during any of the four serial passages (Table 2). Additionally, RT-qPCR analysis showed that there was no decrease in Ct values over the course of passaging, indicating that there was no viral replication occurring. Together, these results demonstrate that both tested concentrations of 0.1% and 0.2% beta-propiolactone effectively inactivated H5N1 virus in allantoic fluid and cell culture media.

We also examined whether treatment of allantoic fluid with 95% ethanol in combination with Qiagen Buffer AVL can inactivate HPAI H5N1 within this fluid. Buffer AVL is the lysis buffer included in the Qiagen QIAamp Viral RNA Mini Kit, which can be used for the extraction of viral RNA from cell-free liquids (23). The protocol involves first adding Buffer AVL to the sample, followed by 95% ethanol (23). Buffer AVL contains high levels of guanidinium thiocyanate, which is a chaotropic agent that disrupts cells and destabilizes macromolecules (24). A study with Ebola virus showed that Buffer AVL on its own reduced virus infectivity, but complete inactivation required the addition of ethanol (25). We also showed that HPAI H5N1 virus in various biological samples, such as milk, urine, and blood, is completely inactivated by Buffer AVL plus 95% ethanol (17). Based on these studies, we tested the combined use of Buffer AVL and 95% ethanol for the inactivation of HPAI H5N1 in allantoic fluid. As seen with the BPL, there was no CPE or increasing levels of viral RNA after serial passaging of Buffer AVL + 95% ethanol treated allantoic fluid (Table 3). These findings indicate that under our experimental conditions, the combination of Buffer AVL and 95% ethanol effectively inactivates H5N1 virus in allantoic fluid, allowing the safe transfer of samples from CL3 to CL2 laboratories for downstream analyses. We acknowledge the caveat that viral RNA may persist even when all viral particles have been inactivated and are no longer infectious.

In conclusion, our study provided protocols for inactivating HPAI H5N1 viruses presented in various samples by various chemical reagents. These protocols ensure biosafety compliance and enable safe handling of inactivated viral materials in lower containment settings without compromising research integrity or public health.
